# Supplementary material for: Analysis of the Efficacy and Risk Factors for Failure of Balloon Dilation for Benign Ureteral Stricture
Source: J Clin Med. 2023 Feb 19;12(4):1655. doi: 10.3390/jcm12041655 (PMC9963490; doi:10.3390/jcm12041655)
Supplement: Supplementary file 1 [file jcm-12-01655-s001.zip › jcm-2165816-supplementary/Supplementary File(s)/supplement Table S1.pdf]

Supplementary Table S1- Demographic and clinical characteristics of patients with balloon dilation after primary balloon dilation and repair surgery

Variable	Upper ureteral				Lower ureteral			
	Primary Failure	Second failure	Secondary Success	Primary Success	Primary Failure	Second failure	Secondary Success	Primary Success
Patients, n	6	4	11	24	5	1	3	29
Mean age (years)	42.67±16.68	38±10.32	31.82±11.64	35.04±14.18	39±12.20	53	52.33±13.07	39.48±15.01
BMI (kg/m2)	23.78±1.98	25.08±1.59	23.54±3.25	23.67±2.45	22.23±1.73	21.3	24.97±2.02	22.73±3.31
Gender, n (%)								
Male	3(50)	4(100)	5(45.45)	17(70.83)	2(40)	0(0)	3(100)	9(31.03)
Female	3(50)	0(0)	6(54.55)	7(29.17)	3(60)	1(100)	0(0)	20(68.97)
Degree of hydronephrosis								
NA	3(50)	1(25)	4(36.36)	6(25)	1(20)	1(100)	0(0)	8(27.59)
Mild/moderate	1(16.67)	2(50)	4(36.36)	14(58.33)	4(80)	0(0)	2(66.67)	17(58.62)
Severe	2(33.33)	1(50)	3(24.28)	4(16.67)	0(0)	0(0)	1 (33.33)	4(13.79)
Preoperative SCr (μmol/L)	69.84±13.06	82.68±7.96	70.74±12.76	77.25±20.28	71.14±24.44	52	130.5±41.91	93.22±77.12
Preoperative BUN（mmol/l）	5.10±1.27	5.66±1.31	4.85±0.75	4.78±1.48	4.63±0.80	3.96	9.38±3.80	6.12±5.02
Preoperative eGFR（ml/min）	99.27±9.75	101.75±14.02	106.15±15.08	110.69±31.27	101.41±23.98	105	74.12±37.45	90.56±29.23
Length of ureteral stricture（cm）	1.33±0.47	1.67±0.94	1.09±0.59	1.10±0.45	0.83±0.24	0.5	0.83±0.24	1.44±0.55
Balloon circumference(mm)	26±2.83	25.5±2.60	24.27±3.25	23.74±2.32	23±1.41	30	24±0	24.14±3.32
Balloon pressure(ATM)	27±3.16	30±0	25.6±4.76	23.67±2.45	24.5±6.95	25	24.33±4.92	27.08±3.20
Dilation Time(min)	3.33±0.75	3.5±0.87	3±0	3±0	3±0	3	3.66±0.94	2.96±0.82
