# Supplementary figures and images for: Analysis of the Efficacy and Risk Factors for Failure of Balloon Dilation for Benign Ureteral Stricture
Source: J Clin Med. 2023 Feb 19;12(4):1655. doi: 10.3390/jcm12041655 (PMC9963490; doi:10.3390/jcm12041655)

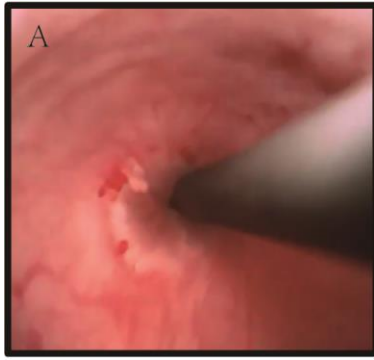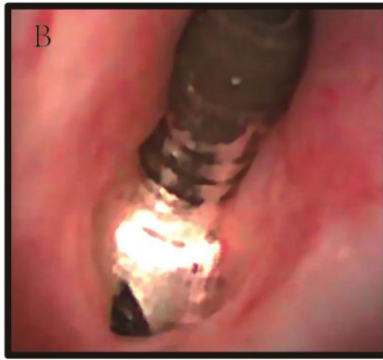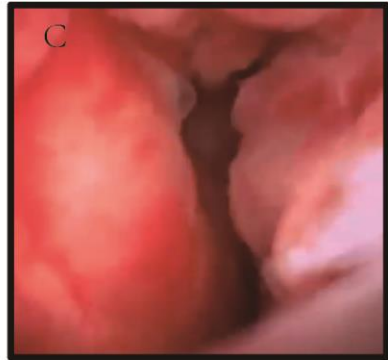

Supplement: Supplementary file 1 [file jcm-12-01655-s001.zip › jcm-2165816-supplementary/Supplementary File(s)/Supplementary Figure S1.pdf]
